# Effect of nutrition education on iodine deficiency disorders and iodized salt intake in south west Ethiopian women: a cluster randomized controlled trial

**DOI:** 10.1186/s12905-020-01126-y

**Published:** 2020-11-16

**Authors:** Agize Asfaw, Tefera Belachew, Taye Gari

**Affiliations:** 1 Department of Public Health, College of Medicine and Health Sciences, Wolkite University, P.O. Box 07, Gubre, Ethiopia; 2 Department of Nutrition and Dietetics, Faculty of Public Health, Jimma University, P.O. Box 378, Jimma, Ethiopia; 3 Department of Public Health and Environmental Sciences, College of Medicine and Health Sciences, Hawassa University, Hawassa, Ethiopia

**Keywords:** Ethiopia, Iodine nutrition, Peer-guided, Iodized salt

## Abstract

**Background:**

Although iodine nutrition status is improving globally, the progress is not uniform throughout the world due to several factors. Among these, poor knowledge, negative attitude and improper practice of iodized salt are the main risk factors for poor iodine nutrition in Ethiopia. This study was aimed to assess the effect of nutrition education intervention on knowledge, attitude and practice (KAP) of iodine deficiency and iodized salt utilization.

**Methods:**

A cluster randomized controlled trial was carried out among 652 women of reproductive age group in southwest Ethiopia. A total of 24 clusters were selected and randomized in to an intervention and control villages. Women in the intervention village received iodine nutrition related education for 6 months; while those in the control village did not receive any education. Baseline and endline data were collected from both groups. Generalized Estimating Equations (GEE) was used to determine the effect of intervention.

**Results:**

A total of 647 (99.2%) participants were successfully involved in the study. In the intervention group the median attendance was 10 out of 12 sessions. Women in the intervention group had shown statistically significant change in knowledge, attitude and practice scores as compared to control one. In multivariable GEE linear model, after adjusting for other background characteristics, the mean difference (95% CI) scores were 8.81 (8.46, 9.16) for knowledge, 3.35 (3.17, 3.54) for attitude and 2.90 (2.74, 3.05) for practice in the intervention arm.

**Conclusions:**

Well designed and community-based iodine nutrition education is an effective strategy to improve the KAP of iodine deficiency disorders and iodized salt utilization.

*Trial registration* PACTR201809544276357 (Retrospectively registered on 14, Sept. 2018).

https://www.pactr.org.

## Background

Iodine is an essential micronutrient that supports different physiological functions in the body. Naturally, our body does not make it, so we need small quantity regularly from outside sources. Running low in iodine intake for long period of time will lead to its deficiency collectively called iodine deficiency disorder (IDD) [1, 2].

Globally, iodine nutrition status is improving from time to time. According to the previous studies, iodine deficient countries declined from 113 in 1993 to 30 in 2013 and only 19 countries remain iodine-deficient in 2017 [3–5]. However; the progress is not uniform throughout the world. Overall, there has been steady progress in developed countries and urban settings. But, there has been minimal progress in rural areas of the developing countries like Africa. Therefore, a great global effort is needed to achieve sustainable elimination of IDD in these iodine deficient areas and maintain the achievement for the decades to come in all countries.

In Ethiopia, eradication of IDD remains a significant challenge due to several factors. From government side: lack of regular enforcement of the legislation, weak regulation and monitoring of the producers and retailers leading to poor salt handling and storage. On the population side, poor knowledge, negative attitude and improper practice of iodized salt are common [6–8].

Despite the efforts made by the Ethiopian government on universal salt iodization for the last decade, there was still unacceptably high goiter prevalence (54.6%) indicating the presence of severe chronic iodine deficiency in the area. Moreover, 63.5% of the salt consumed by the family was insufficiently iodized and 17.4% contained no iodine at all [9]. These are striking findings for a country like Ethiopia where universal salt iodization (USI) is being implemented.

On the other hand, human behavior is a complex process influenced by many factors related to knowledge, attitude, norms, cultural practices, personal preferences and level of efforts needed to change that behavior [10, 11]. However; as recommended by several previous studies, public education and awareness raising campaigns are useful tools to bring behavior change and address the challenges of IDD prevention and control program [12–22].

In current study area, there is scarcity of evidence on the effect of interventions targeting the knowledge, attitude and practices (KAP) of households related to iodine nutrition. In this study we set out to document the effect of community-based and peer-guided iodine nutrition education intervention in improving knowledge of IDD, attitude towards and practice of iodized salt in settings where IDD is endemic and iodized salt consumption is poor.

To achieve the objective, women of reproductive age group were selected as a target group, because women and children are highly vulnerable to IDD and relatively representative of the study population in IDD surveillance [23]. In addition, women are mainly responsible for household meal preparation using iodized salt.

## Methods

### The study area

This study was conducted in Dawro Zone, southwest part of Ethiopia. The zone has five districts and one town administration with a total land area of 4437 km^2^. It has three different agro-ecological zones; high land with altitude range of 2300–3200 m above sea level (masl), middle land between 1500 and 2300 masl and low land below 1500 masl. The mean annual temperature and rainfall ranges from 15.1 to 27.5 °C and 1200–1800 mm respectively [24, 25].

The total population and health service coverage of the zone in 2016 was 702,517 and 93% respectively. Women of reproductive age group (15–49 years) accounted for 23% of the total population of the zone [26]. IDD is endemic and iodized salt consumption is poor in the study area [9, 27].

### Study design

Cluster randomized controlled trial was carried out for six months from January to June 2017. The unit of randomization was households served by health development armies (villages having 27–30 households). A health development army (HDA) in Ethiopia is defined as a network of women volunteers in the same village organized to promote health, prevent disease through community participation and empowerment. The HDA facilitate identification of local problems that hinder families from utilizing key maternal, neonatal and child health services and to come up with locally acceptable strategies to address the problem. They are also responsible frontline actors in discussing preventive health issues including health education and mobilize the community through different campaigns.

## Participants

### HDA inclusion and exclusion criteria

This trial was conducted in 24 randomly selected HDAs (clusters). The clusters were stratified in to highland and lowland by expecting knowledge, attitude and practice (KAP) differences in IDD and iodized salt utilization between the dwellers of these two locations. As indicated by previous studies, highland areas are more at risk of IDD [28, 29]. Therefore, people living in these areas have more exposure to visible sequale of IDD like goiter and could have better knowledge about it. All the selected clusters in highland and lowland kebeles with a minimum inter-cluster distance of 9kms (to minimize information contamination) were included in the trial. Middle land clusters located at the boarder of lowland and highland (having no clear boundary) were excluded.

### Participant inclusion and exclusion criteria

Individual participants were all women or reproductive age group (15–49 years) who were primarily responsible for meal preparation. Women who refused to give verbal consent, those who were mentally ill; those who had severe learning difficulties or planned to leave the area within next one year were excluded.

### Randomization

The study zone had five districts and one town administration. Two of the districts were selected by simple random sampling (SRS) and the town administration was included. The list of kebeles (the smallest administrative unit in the district) as a sampling frame was obtained from each district and the town administration bureau.

**Fig. 1:**
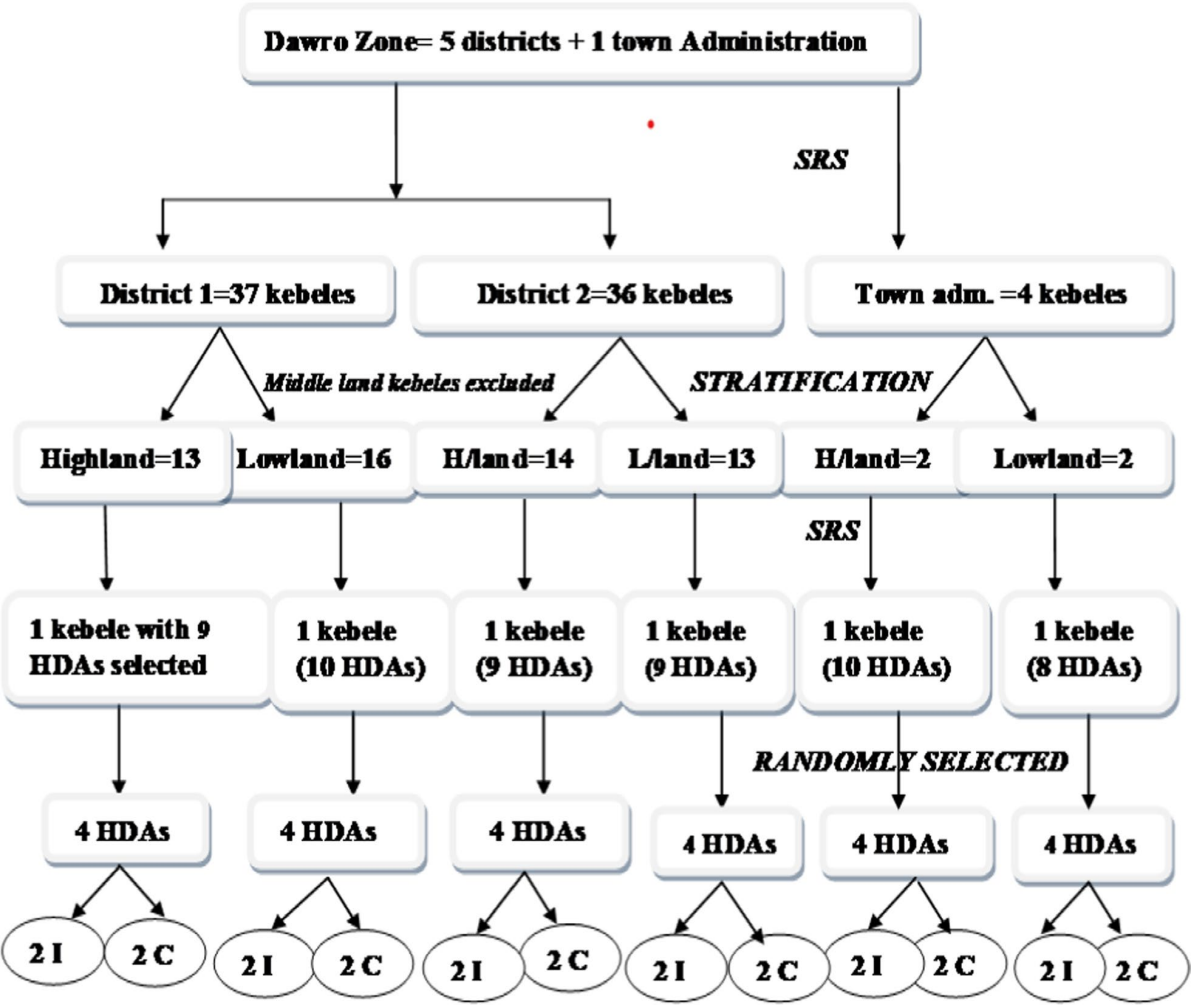
Schematic presentation of sampling procedure in southwest Ethiopia, 2017

The kebeles were stratified in to highland and lowland. Accordingly, 29 highland and 31 lowland kebeles were identified, sequentially numbered and three of them randomly drawn from each stratum. In the six selected kebeles there were a total of 55 HDAs (clusters). Among them, four clusters were randomly selected from each Kebele (4*6 = 24clusters). Finally, two of the clusters were randomly assigned to intervention or control arm by a supervisor (health officer with BSc degree) who was unaware of the study-arm assignments in each stratum. Due to the nature of intervention, blinding of the participants was not applied. The schematic presentation of the sampling procedure was summarized in (Fig. 1).

### Intervention modalities

The intervention was targeted at the cluster level. Clusters were health development armies containing 27–30 households in the village. From each household a women of reproductive age group who was primarily responsible for household meal preparation was selected to participate in the trial.

### Tool development

To execute the trial, first educational and training tools were prepared. The tools contain clear message on iodine, its source, benefits of iodized salt, how to handle and use iodized salt, how to store it and the consequences of not using iodized salt. To prepare these tools, we referred available resources from World Health Organization (WHO), United Nations children’s fund (UNICEF), Iodine Global Network (IGN), Ethiopian Demographic and Health Survey (EDHS), National micro-nutrient guide lines and adapted to local context and target groups [23, 30–34].

### Health Development Army members selection for training

Two HDA members who fulfill the criteria (Volunteer, 10th grade completed, fluent in speaking local language, active participant in the group, stable and live in the area for the next one year) were selected from each intervention site. Five days training supported by demonstration and posters was given by principal investigator (PI). A copy of the teaching material was also given for each TOT (training of trainer) members.

### Nutrition education for intervention groups

For intervention groups, a 6 month iodine nutrition education was given by using the prepared materials. Members of HDA who actively completed five days training handled the sessions. The schedule for the session was twice every month for six consecutive months. In each 1 h.

Education session, two-way process of sharing message, active involvement of the participants, freely exchange of ideas and demonstration of iodized salt was practiced.

### Control groups

On the contrary to the intervention clusters, the control clusters did not participate in any nutrition education session. They continued with their routine health care services of the ministry of health (MOH) as usual. The MOH routine health care package for the frontline health workers in Ethiopia includes 16 components. These components can be categorized in to four major areas: family health (family planning, maternal and child health, nutrition and vaccination services), disease prevention and control (HIV/AIDS and STI, tuberculosis, malaria and first aid cares), hygiene and sanitation (promotion of sanitary latrines, waste disposal management, water supply, food hygiene and safety, control of insects and rodents, personal hygiene and healthy home environment services) and health education and communication [35].

### Supervision, endline data collection and measurement

The sessions were monitored by PI and/or assigned supervisor every month. At the end of each three month, there was review meeting in which all members including the supervisor present their work in the presence of PI. The purpose of this three months review meeting was for monitoring and process evaluation to ensure the sessions were being implemented as planned or need any adjustment. At the end of intervention period (after six months), data were collected from both groups (intervention and control) using the same data collectors and questionnaire that was used at the baseline.

All factors and tools used were made similar for both arms except the intervention. This ensured that the observed change in intervention arm was reasonably attributed to the intervention. The change in KAP (increment in knowledge, change in attitudes or reduction of risky behaviors, and improvement of practice) was measured by comparing the baseline data with end line findings.

### Deviations from the protocol

Generally, the intervention was carried out as planned. However, there were some deviations from the protocol. Initially, the intervention was planned for 9 months, but we believe this is too long to see the effect of intervention and reduced to six months. As this is one of the unfunded studies, we could not be able to measure urinary iodine concentration of participants and failed to assess nutritional status as a secondary outcome. The last deviation from the study protocol was that the study lagged seven months behind the schedule because of the political instability in the country that interfered data collection.

### Outcomes

The outcomes were change in mean score of the three composite variables (knowledge, attitude and practice) about IDD and iodized salt after intervention. The secondary outcome, according to our plan, was change in iodine nutrition status of participants after intervention. Unfortunately, this was not done due to resource limitations.

### Sample size determination

The sample size was calculated using GPOWER 3:0 Software, by taking into account the intracluster correlation coefficient (ICC), the average cluster size, the expected effect size, and the power of the study. The ICC was assumed to be 0.04 from previous studies [36, 37], total number of clusters 24 with a minimum of 27 participants in each cluster and the expected effect size of 0.29. The effect size was estimated from the previous studies conducted in south and northwest Ethiopia [38, 39]. The study in southwest Ethiopia reported about 44.7% respondents had good knowledge and 42.6% had positive attitude towards consumption of iodized salt [38]. Similarly the northwest Ethiopian study reported about half of the households’ (49.8%) added salt at the beginning and middle of food preparation (inappropriate practice). This means, the rest 50.2% added at the end of cooking process (appropriate practice) [39].

Therefore, most likely the current study zone might have similar proportions and we planned to increase good knowledge from 44.7 to 74%, positive attitude from 42.6 to 72% and appropriate practice from 50.2 to 79%. With these assumptions a power of 80% was anticipated to detect a difference in KAP scores between the two groups with *α* err prob. of 0.05. Finally, 10% expected non-response rate/drop-out was added to get 652 as the sample size for the trial.

### Data collection

Training was given for twelve data collectors (health extension workers) on how to use the questionnaire, ethical issue, revisiting closed houses and their work relationship with the supervisor for 3 days. The questionnaire was pretested on non-selected but similar adjacent kebeles. A week after pretest, baseline data were collected from both intervention and control clusters by face to face interview using structured and modified questionnaire. The same tool and data collectors were used to collect endline data after 6 months of intervention.

### Instrument validity

For face validity, 32 households (5% of the sample) were interviewed and each questionnaire was completed by data collectors. The completed questionnaire was collected and evaluated by the principal investigator, co-investigators and a statistician. For content validity, an expert of three specialists in endocrinology, nutrition and biostatistics examined the initial questionnaire. The experts were asked to comment on individual items in relation to the accuracy and content. Finally, items were modified slightly based on the findings from pretest and the expert reviews.

In addition, Cronbach’s *α* was determined for internal consistency (Cronbach’s *α* = 0.93, 0.77 and 0.71 for knowledge, attitude and practice respectively). Addition of iodized salt at the end of cooking process, not washing the salt before use and not exposing it to sunlight are some of the recommended practices of iodized salt. Convergent validity was checked by observing the practice of women against these recommended practices during demonstration sessions.

### Measurement

Data were collected using a series of 16 questions about knowledge of IDD and iodized salt and eight questions about practice of iodized salt. In each case a correct response was given a score of one, whereas an incorrect response was given a score of zero. Then, knowledge and practice indices were produced by adding individual answers across items.

Similarly, data on attitude about iodized salt and goiter were collected using a series of seven questions. Each question had three options (yes, no or don’t know) and a three-point Likert scale (agree, disagree or neutral). The responses were dichotomized in to “yes” or “no” and “agree” or “disagree” as there was no response on third options (“don’t know” and “neutral”). A score of one was given for responses that defined positive attitude and a score of zero was given for negative ones. An index for attitude was produced after summing individual answers across items.

Finally, for all the three indices (knowledge, attitude and practice scores), we computed the difference by subtracting the baseline value from the endline. These differences were used as response variables for the subsequent analysis.

### Data processing and analysis

Data were doubly entered into Epi Info version 3.5.3, cleaned and analyzed using SPSS version 21 (SPSS Inc. Chicago, USA). Descriptive statistics were summarized using frequency and proportions. The mean difference in KAP scores between intervention and control groups was tested using independent sample t-test. Principal component analysis (PCA) was used to construct a wealth index from 20 household fixed asset such as presence of latrine, source of drinking water, possession of television, radio, mobile telephone, availability of separate kitchen from living house, domestic animal and land possessed in hectare. These variables were dichotomized and coded ‘1’ for the household possessing the asset and ‘0’ for the rest. Finally, the factor scores ranked ordered into three relative measures of socio-economic classes (poor, medium and rich).

For this study, the primary outcome measures were composite variables (knowledge, attitude and practice scores) determined by summing all responses across items and subtracting the baseline sum from the endline to get the difference. These differences in scores were taken as an outcome variable for each.

As mentioned above, there were two measurements on the same subject (baseline and endline). Since repeated observations within one subject are considered not independent of each other, the standard linear regression could not allow for the repeated measures and could underestimate the standard error. In this case, the Generalized Estimating Equations (GEE) was used to extend the generalized linear model to allow for analysis of repeated measurements. GEE helps to correct for the expected within-subject correlations [40]. Therefore, after checking all assumptions including multicolinearity (maximum VIF = 1.240), we fitted generalized linear models (GEE).

First, we run bivariate analysis for each outcome variable. Then, to control for confounding and adjust for within cluster correlation of measurements, all variables with a *p* value ≤ 0.2 in the bivariate analysis were fitted into the model for multivariable analysis. All analysis was conducted according to intention-to treat principles and a *p* value of 0.05 or less was considered statistically significant.

## Results

### Socio-economic and outcome variables at baseline

Baseline data collected before the start of the intervention in 2016 showed that the two groups were comparable for both socioeconomic and outcome variables, except for age group and wealth category. Relatively higher numbers of participants with advanced age category (35–49 years) were found in the intervention group (37.1% vs. 17.8%). The numbers of participants with poor and medium wealth status were higher in control (44.5% vs. 34.7%) and intervention (25.2% vs. 16.3%) groups respectively. All the participants of this study were women of reproductive age group (15–49 years) in southwest Ethiopia (Table 1).

**Table 1:** Description of socio-demographic and outcome variables at baseline in intervention and control groups, southwest Ethiopia, 2016

Variable	Category	Groups	
		Intervention (n = 326) Number (%)	Control (n = 326) Number (%)
Age category in years	15–24	69 (21.2)	93 (28.5)
	25–34	136 (41.7)	175 (53.7)
	35–44	85 (26.1)	42 (12.9)
	45–49	36 (11.0)	16 (4.9)
Family size (number)	≤ 5	198 (60.7)	218 (66.9)
	> 5	128 (39.3)	108 (33.1)
Educational status	Informal	195 (59.8)	190 (58.3)
	Formal	131 (40.2)	136 (41.7)
Marital status	Married	265 (81.3)	257 (78.8)
	Single^θ^	61 (18.7)	69 (21.2)
Geographic location	Highland	164 (50.3)	162 (49.7)
	Lowland	162 (49.7)	164 (50.3)
Wealth index	Poor	113 (34.7)	145 (44.5)
	Medium	82 (25.2)	53 (16.3)
	Rich	131 (40.2)	128 (39.3)
Knowledge score (5.22 ± 2.89)^µ^	Poor	187 (57.4)	209 (64.1)
	Good	139 (42.6)	117 (35.9)
Attitude score (3.36 ± 2.01)^µ^	Unfavorable	213 (65.3)	210 (64.4)
	Favorable	113 (34.7)	116 (35.6)
Practice score (4.26 ± 1.53)^µ^	Inappropriate	199 (61.0)	189 (58.0)
	Appropriate	127 (39.0)	137 (42.0)

Single^θ^-not married, separately living, divorced and widowed

^µ^(Mean ± SD)

### Response rate and attendance

**Fig. 2:**
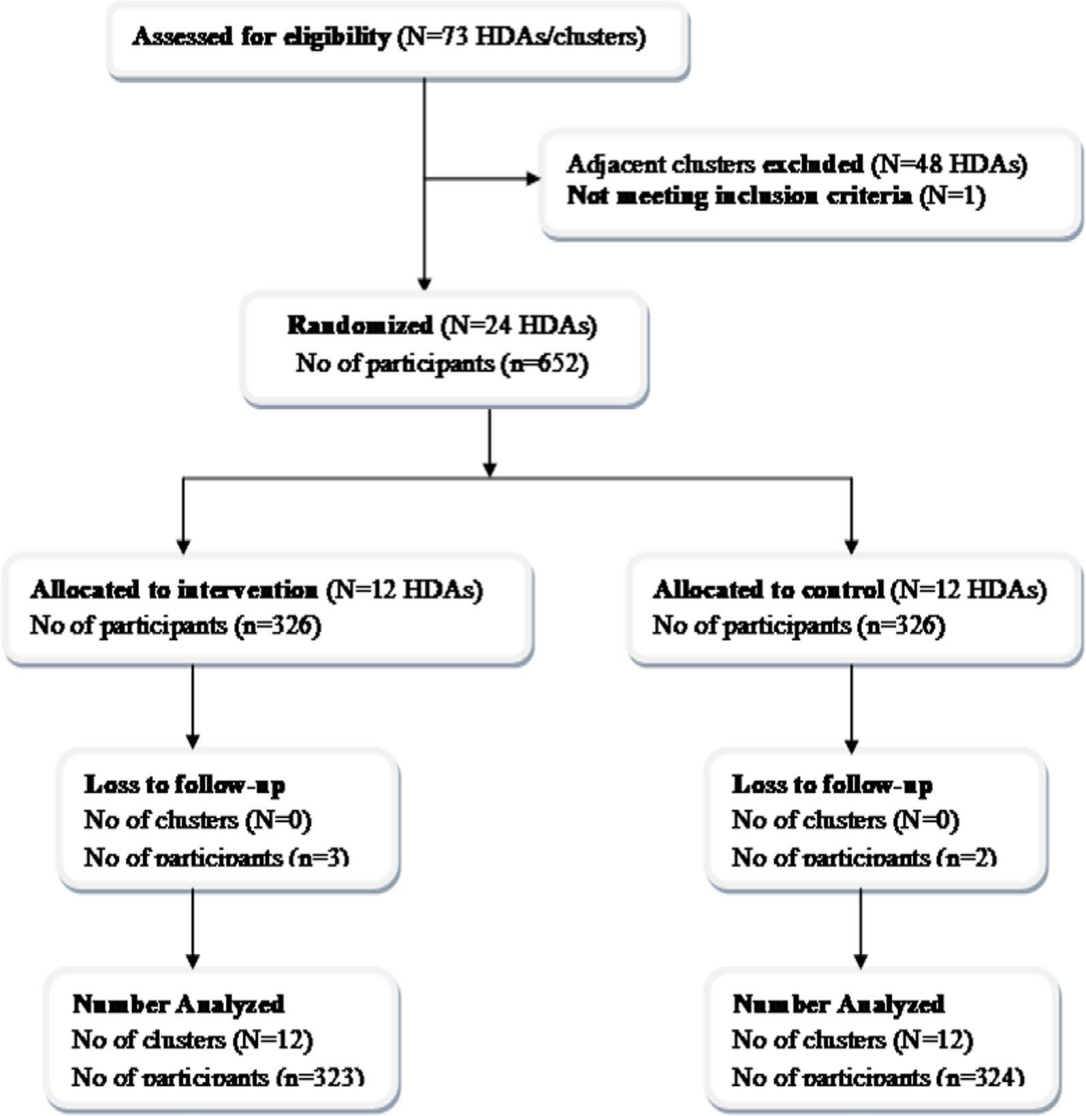
Study profile for iodine nutrition education intervention among women of reproductive age in south west Ethiopia, 2017

A total of 647 (99.2%) participants were successfully surveyed at the endline. In the intervention group, the median attendance was 10 (ranging from 4 to 12 sessions). All participants in the intervention group attended the first session, but three (0.9%) of them dropped out during the intervention period. Similarly, two (0.6%) participants were disappeared from the control group. The overall participant flow had been summarized using CONSORT flow diagram (Fig. 2).

### Outcomes and estimation

Scores in knowledge about IDD, attitude towards and practice of iodized salt were analyzed for change between groups after six months nutrition education intervention. To appreciate the change, we calculated the mean difference between the two groups (intervention vs control) with the corresponding precision (95% CI) for each of the primary outcomes (knowledge, attitude and practice scores) after adjustment for the baseline covariates. The adjusted effect measures were considered as the main results.

Women in the intervention group had shown statistically significant change in knowledge, attitude and practice scores as compared to control one. The mean difference (MD) ± standard deviation (SD) for women in the intervention group was (8.82 ± 3.56) for knowledge, (3.00 ± 2.15) for attitude and (3.11 ± 1.67) for practice scores respectively. The differences in score for all three outcome variables were statistically significant with knowledge (β = 8.81; 95% CI: 8.46, 9.16), attitude (β = 3.35; 95% CI: 3.17, 3.54) and practice (β = 2.90; 95% CI: 2.74, 3.05). For the control groups such differences were negligible for all the outcome variables as indicated in Table 2.

**Table 2:** Effect of nutrition education intervention on knowledge, attitude and practice of iodine deficiency and iodized salt consumption in southwest Ethiopian Women

Outcome variables	Groups	Baseline Mean (SD)	Endline Mean (SD)	Difference Mean (SD)	Adjusted effect (DoD), (95% CI)
Knowledge score	Intervention	5.36 (2.94)	14.20 (2.00)	8.82 (3.56)	8.81 (8.46, 9.16)
	Control	5.08 (2.83)	5.24 (3.12)	0.14 (0.86)	Ref
Attitude score	Intervention	3.42 (2.03)	6.43 (0.92)	3.00 (2.15)	3.35 (3.17, 3.54)
	Control	3.30 (1.99)	3.12 (1.49)	-0.19 (2.92)	Ref
Practice score	Intervention	4.21 (1.56)	7.32 (0.84)	3.11 (1.67)	2.90 (2.74, 3.05)
	Control	4.32 (1.50)	4.45 (1.51)	0.12 (0.78)	Ref

*SD* standard deviation, *DOD* difference of difference

To identify independent factors associated with the endline-baseline difference of the differences in the mean KAP scores, we employed multivariable Generalized Estimating Equations (GEE). Variables included in the model were: age, family size, educational status, geographic location, wealth index, availability of iodized salt in nearby shop/market and the intervention.

Out of all other variables, intervention was the only independent predictor of all three outcome variable scores (knowledge, attitude and practice). Among the remaining variables, only wealth index was significantly associated with the knowledge score. On the other hand, family size, educational status and wealth index were significantly associated with attitude score and geographic location and availability of iodized salt in nearby shop were significantly associated with practice score.

All the three outcome variable scores were increased on average nearly by nine points for knowledge (β = 8.81, 95% CI = 8.46, 9.16), by three points for attitude (β = 3.35, 95% CI = 3.17, 3.54), and practice (β = 2.90, 95% CI = 2.74, 3.05) each in the intervention arm. Wealth index and knowledge score were inversely related (β = − 0.499, 95% CI = − 0.947, − 0.050). For a unit increase in family size, the average attitude score increased by 0.538 (β = 0.538, 95% CI = 0.106, 0.970). Similarly, attitude score increased as educational level increases (β = 0.573, 95% CI = 0.174, 0.971) and for the poor wealth category (β = 0.695, 95% CI = 0.249, 1.142). On t he other hand, practice score decreased (β = − 0.567, 95% CI = − 0.760, − 0.375) as the availability of iodized salt in nearby shop/market decreased and for the highland dwellers (β = − 0.314, 95% CI = − 0.512, − 0.117) (Table 3).

**Table 3:** Generalized estimating equations predicting baseline-endline difference of the differences in knowledge, attitude and practice of iodine deficiency disorder and iodized salt among women in Southwest Ethiopia, 2016–2017

Predictors	Knowledge			Attitude			Practice		
	β (SE)	95% CI		β (SE)	95% CI		β (SE)	95% CI	
		Lower	Upper		Lower	Upper		Lower	Upper
Nutrition education	8.809** (0.178)	*8.459*	*9.158*	3.352** (0.094)	*3.168*	*3.535*	2.895** (0.081)	*2.737*	*3.054*
Age in year	− 0.010 (0.015)	− 0.038	0.019	− 0.004 (0.013)	− 0.029	0.021	0.005 (0.008)	− 0.010	0.020
Family size	− 0.250 (0.225)	− 0.692	0.191	0.544* (0.220)	*0.113*	*0.975*	0.161 (0.114)	− 0.062	0.384
Educational status	0.233 (0.212)	− 0.182	0.647	0.579* (0.203)	*0.180*	*0.977*	− 0.095 (0.103)	− 0.297	0.106
Geographic location	− 0.395 (0.209)	− 0.804	0.014	0.154 (0.196)	− 0.230	0.538	− 0.315* (0.100)	*0.511*	− *0.119*
Wealth index									
Medium	− 0.190 (0.283)	− 0.744	0.364	0.240 (0.265)	− 0.278	0.759	− 0.278 (0.150)	− 0.572	0.017
Poor	− 0.511* (0.230)	− *0.962*	*0.059*	0.700* (0.229)	*0.252*	*1.148*	− 0.093 (0.106)	− 0.300	0.114
Iodized salt^+^	− 0.284 (0.210)	− 0.696	0.128	− 0.164 (0.202)	− 0.560	0.231	− 0.612** (0.099)	*0.807*	*0.417*

Italics indicate different levels of significance

Reference categories: Nutrition education (control), age (continuous variable), family size (≤5), educational status (formal), geographic location (lowland), wealth index (rich), iodized salt (available)

**Significant at P < 0.0001

*Significant at P < 0.05

^+^Iodized salt, not available in nearby shop/market

## Discussion

Despite global improvement in iodine nutrition status, achieving sustainable elimination of its deficiency remained a great challenge in Ethiopia. Consumption of iodized salt, the most widely recommended global strategy to control and eliminate iodine deficiency, had several limitations and gaps in the country. Among these, knowledge gap, misperceptions and improper practices of iodized salt are at the top list [18–21].

This community based cluster randomized controlled trial study was designed and implemented iodine nutrition education intervention to address such problem in southwest Ethiopia. Before the study, baseline survey was conducted and identified the existing levels of KAP concerning IDD and iodized salt and the gap.

At the beginning of the study, there was no significant difference for KAP scores about IDD and iodized salt in both intervention and control groups. Approximately half of the women in both groups scored below mean at baseline. However; as summarized in Table 2 and 3, for all three outcome variable scores there was significant difference between the two groups after six months of nutrition education intervention.

The average knowledge score approximately tripled for intervention arm at the endline. Similar results were observed in studies conducted in India to find out the impact of health education intervention on awareness and consumption of iodized salt in school children [14] and a randomized controlled trial conducted in Iran to evaluate the effectiveness of educational program on the iodine nutrition status of pregnant women [19].

Regarding the attitude and practice scores, the mean differences were 3.35 and 2.90 respectively at the endline. This means, on average, the scores tripled for both attitude and practice after the nutrition education intervention. The findings were supported by previous studies conducted and identified the positive impact of education and awareness raising program to increase the iodized salt consumption in Pakistan [14], India [14] and Iran [19].

As indicated in multivariable analysis, factors such as educational status, wealth status, geographic location and availability of iodized salt in nearby shop/market should be considered before designing education sessions. In this study, participants with poor wealth category and informal education had shown favorable attitude towards iodized salt. On the other hand, being in poor wealth category and living in highland area were negatively related with knowledge and practice scores respectively.

According to the finding of this study, the effect of nutrition education was similar across women with different educational status for knowledge component. However, for poor women who had no formal education, the sessions should focus on attitude component. In addition, one should ensure the accessibility of iodized salt before designing educational sessions on practice as participants who had no access to iodized salt within less than 30 min walking distance had shown poor practice of iodized salt.

Generally, the outcomes of this study were clear indications of the effectiveness of iodine nutrition related education intervention in improving the knowledge about IDD and iodized salt, changing misperception and inappropriate practice of iodized salt in the study population.

The findings of this study have wide practical implications. Although the government of Ethiopia has been implementing iodization of salt as an intervention strategy for the past one decade, there are still differences in the way households understand the program and their uptake of it. The findings also showed that giving enhanced education through the existing government structure including the frontline actors and health development army will significantly contribute to the reduction of IDD in Ethiopia.

Almost all sessions were peer-guided; interactive with free exchange of ideas and some of them were demonstration based, which could be considered as strength of this study. The fact that the questionnaire was filled based on self-report could be acknowledged as a limitation as it might induce some social desirability bias. However, efforts were made to probe the participants and explain the purpose of the study and we believe that it may not affect the validity. The other limitation could be our failure to assess nutritional status of participants. Even though we planned to measure nutritional status as a secondary outcome, we couldn’t be able to do this because of limited resources. However; failure to do this, may not affect our findings and conclusion as this was not our primary objective.

## Conclusions

Despite the complex nature of human behavior, a well-designed, community-based and peer-guided iodine nutrition education is an effective tool to improve knowledge about IDD and to change misperception and inappropriate practice of iodized salt in settings where IDD is endemic and iodized salt consumption is poor.

## Data Availability

All data used and/or analyzed during the current study are available from the corresponding author on reasonable request.

## Abbreviations

CONSORT: Consolidated Standards of Reporting Trials
EDHS: Ethiopian Demographic and Health Survey
GEE: Generalized estimating equations
HAD: Health development army
HEW: Health extension worker
IDD: Iodine deficiency disorders
IGN: Iodine Global Network
KAP: Knowledge, attitude, practice
MOH: Ministry of Health
PCA: Principal component analysis
PI: Principal investigator
SRS: Simple random sampling
UNICEF: United Nations Children’s Fund
USI: Universal salt iodization
VIF: Variance inflation factor
WHO: World Health Organization

## References

[1] Aburto N, Abudou M, Candeias V, Wu T. Effect and safety of salt iodization to prevent iodine deficiency disorders: a systematic review with meta-analyses. WHO eLibrary of Evidence for Nutrition Actions (eLENA). World Health Organization, Geneva. 2014; https://apps.who.int/iris/bitstream/10665/148175/1/9789241508285_eng.pdf.

[2] Hernando VU, Anilza BP, Hernan STC (2015). Iodine deficiency disorders. Thyroid disorders. Therapy.

[3] Andersson M, Karumbunathan V, Zimmermann MB (2012). Global iodine status in 2011 and trends over the past decade. J Nutr.

[4] Pearce EN, Andersson M, Zimmermann MB (2013). Global iodine nutrition: where do we stand in 2013? Zurich, Switzerland. Thyroid.

[5] Branca F, Vollmer J, Zimmerman M. Eliminating iodine deficiency disorders by 2020. Zurich, Switzerland. 26 May 2017. https://www.gainhealth.org/knowledge-centre/toward-elimination-iodine-deficiency-disorders-2020/D. Accessed on 25 Oct 2017.

[6] Gebretsadikan TM, Troen AM (2016). Progress and challenges in eliminating iodine deficiency in Ethiopia: a systematic review. BMC Nutr.

[7] Girma K, Nibret E, Gedefaw M (2014). The status of iodine nutrition and iodine deficiency disorders among school children in Metekel zone, Northwest Ethiopia. Eth J Health Sci.

[8] Gebremariam HG, Yesuf ME, Koye DN (2013). Availability of adequately iodized salt at household level and associated factors in Gondar Town, Northwest Ethiopia. ISRN Public Health.

[9] Asfaw A, Belachew T (2020). Magnitude of iodine deficiency disorder and associated factors in Dawro zone, southwest Ethiopia; the hidden hunger: a cross-sectional study. BMC Nutr.

[10] Kelly MP, Barker M (2016). Why is changing health-related behavior so difficult?. Public Health.

[11] Johnson MJ, May CR (2015). Promoting professional behavior change in healthcare: what interventions work, and why? A theory-led overview of systematic reviews. BMJ Open.

[12] Macias YF, Glasauer P. Guidelines for assessing nutrition-related knowledge, attitudes and practices. Food and Agriculture Organization of the United Nations, Rome, 2014. https://www.fao.org/docrep/019/i3545e/i3545e.pdf.

[13] Lowe N, Westaway E, Munir A (2015). Increasing awareness and use of iodized salt in a marginalized community setting in North-West Pakistan. J Nutr.

[14] Ansari MA, Khan Z (2016). Impact of health education intervention on consumption of iodized salt in school children in Aligarh, India. Int J Commun Med Public Health.

[15] Garnweidner-Holme L, Aakre I, Lilleengen AM, Brantsæter AL, Henjum S (2017). Knowledge about iodine in pregnant and lactating women in the Oslo area, Norway. Nutrients.

[16] Lucas CJ, Charlton KE, Brown L, Brock E, Cummins L (2014). Antenatal shared care: Are pregnant women being adequately informed about iodine and nutritional supplementation?. Aust N Z J Obstet Gynaecol.

[17] Naghashpour M, Shakerinejad G, Reza M, Hajinajaf LS, Jarvandi F (2014). Nutrition education based on health belief model improves dietary calcium intake among female students of junior high schools. J Health Popul Nutr.

[18] Mehran L, Nazeri P, Delshad H, Mirmiran P, Mehrabi Y, Azizi F (2012). Does a text messaging intervention improve knowledge, attitudes and practice regarding iodine deficiency and iodized salt consumption?. Iran Public Health Nutrition.

[19] Amiri P, Hamzavi Zarghani N, Nazeri P (2017). Can an educational intervention improve iodine nutrition status in pregnant women? A randomized controlled trial. Thyroid.

[20] Ahmed AE, Elnour SA, Ahmed YM (2017). Knowledge and attitude of population towards iodized salt in Shendi locality river Nile state in Sudan. Eur Sci J.

[21] O’Kane S, Pourshahidi L, Farren K, Mulhern M, Strain J, Yeates A (2016). Iodine knowledge is positively associated with dietary iodine intake among women of childbearing age in the UK and Ireland. Br J Nutr.

[22] Kidane T, Woldegebriel A (2006). Prevalence of iodine deficiency disorder in a highland district in Tigray: brief communication. Ethip J Health Dev.

[23] World Health Organization (2007). Assessment of iodine deficiency disorders and monitoring their elimination: A guide for program managers.

[24] Alemayehu. A. Enset Value Chain, the Case of Dawro Zone, Southern Nations Nationalities and Peoples Regional State, Ethiopia. International Journal of African and Asian Studies, Vol.30, 2017. ISSN 2409–6938. https://iiste.org/Journals/index.php/JAAS/ article/view File/35383/36403

[25] FDRE. The development study on the strengthening agricultural marketing system in southern nation’s nationalities and peoples region. Final report, November 2012. PP.2.2. Accessed 25, March 2018.

[26] Central Statistical Agency [Ethiopia]. 2014. Ethiopia Mini Demographic and Health Survey 2014. Addis Ababa, Ethiopia

[27] Workie SB, Abebe YG, Gelaye AA, Mekonen TC (2017). Assessing the status of iodine deficiency disorder (IDD) and associated factors in Wolaita and Dawro Zones School Adolescents, southern Ethiopia. BMC Res Notes.

[28] Abuye C, Berhane Y, Ersumo T (2008). The role of changing diet and altitude on Goitre prevalence in 5 regional states in Ethiopia. East Afr J Public Health.

[29] Ahmed M, Zama SY, Nagarajarao V, Khan MA (2014). Iodine deficiency in children: a comparative study in two districts of south-interior Karnataka. India J Fam Community Med.

[30] Central Statistical Agency [Ethiopia] and ORC Macro EDHS 2011. CSA, Addis Ababa, Ethiopia and Calverton, Maryland, USA; 2012. https://dhs.program.com/pubs/pdf/FR255/FR255.pdf.

[31] Iodine Global Network. Global Scorecard of Iodine Nutrition in 2017 in the general population and in pregnant women (PW). IGN: Zurich, Switzerland. 2017. Available at: https://www.ign.org/cm_data/IGN_Global_Scorecard_AllPop_and_PW_May2017.pdf. Accessed on 25, Oct 2017.

[32] UNICEF, IGN. Technical Working Group Meeting on Research Priorities for the Monitoring of Salt Iodization Programs and Determination of Population Iodine Status. New York: UNICEF; 2016. Found at https://www.unicef.org/nutrition/files/UNICEF_IGN_Iodine_Technical_Consultation_2015.pdf.

[33] FMOH. National guideline for control and prevention of micronutrient deficiencies. Ethiopia, June 2004. Date accessed, 16 April 2016.

[34] WHO. Elimination of iodine deficiency disorders. A manual for health workers. EMRO Technical Publications Series 35; 2008. https://applications.emro.who.int/dsaf/dsa928.pdf

[35] Assefa Y, Gelaw YA, Hill PS (2019). Community health extension program of Ethiopia, 2003–2018: successes and challenges toward universal coverage for primary healthcare services. Global Health.

[36] Taljaard M, Grimshaw JM (2014). Concept, characteristics and implications of cluster randomization. Clin Invest.

[37] Francis NA, Butler CC, Hood K, Simpson S, Wood F, Nuttall J (2009). Effect of using an interactive booklet about childhood respiratory tract infections in primary care consultations on reconsulting and antibiotic prescribing: a cluster randomized controlled trial. BMJ.

[38] Haji Y, Abdurahmen J, Paulos W (2016). Knowledge and perception of Consumption of iodized salt among food handlers in Southern Ethiopia. SAGE J..

[39] Abebe Z, Gebeye E, Tariku A (2017). Poor dietary diversity, wealth status and use of un- iodized salt are associated with goiter among school children: a cross-sectional study in Ethiopia. BMC Public Health.

[40] Twisk J (2003). Applied longitudinal data analysis for epidemiology: a practical guide.

